# Book Reviews

**Published:** 1800-09

**Authors:** 


### CRITICAL RETROSPECT
OF
### MEDICAL AND PHYSICAL LITERATURE,
[ FOREIGN AND DOMESTIC. ]

*Obfervations on the Cow-Pox, by* WILLIAM WOODVILLE, M. D, Phyfician to the Small-Pox and Inoculation Hofpitals. 8vo. pp. 43. London, W. Phillips.

WHEN the Vaccine Inoculation began to engage the attention of the public, it was immediately perceived by its nature, that two very important pillars of its temple refted on the abfence of eruptions, and the impoffibility of the contagion being communicated, except by infertion of the virus, or inoculation. The inconvenience arifing from thefe fources had always been confidered as fuch formidable objections to the Small-pox, that very expenfive eftablifhments had at that time been begun on the continent, with a view to exterminate it altogether.* When Dr. Jenner therefore, found one part of his immortal difcovery to appear defective in this refpect in the vicinity of the metropolis, and in thofe patients particularly who were inoculated with matter fent from thence, and fcarcely any others, it was impoffible for him, in his fecond publication on the fubject, to avoid offering fome conjecture on the caufe of this diverfity. In doing this, he *criminated* no man; who indeed could criminate fuch a character as Dr. Woodville? We are therefore much hurt to obferve, that Dr. W. fhould find himfelf "unable to avoid a certain degree of *recrimination*, which attaches to a man, for whom he has long entertained a friendly regard, and to whom the public is under the great obligation of having been made acquainted with a difcovery, which promifes the moft important benefits to fociety." There is a gentle mildnefs of temper, in which the caufe of truth delights; but which we do not find fo confpicuous in the firft part of this pamphlet as we expected. With refpect to the refutation of Dr. Jenner's conjecture, the public will judge for themfelves when they have read Dr. W's letter.

The following paffage, we apprehend, admits every thing that Dr. Jenner fuppofed:—"Although I differ in opinion from Dr. Jenner in not imputing the puftular eruptions, produced in the cafes at the hofpital, to any adulteration of the vaccine matter employed in the inoculations, yet I readily admit that they have been and ftill continue to be the effect of fome adventitious caufe, independent of the Cow-pox,

"This

* See Number XVII. p. 90.

"This will clearly appear from the following obfervations, which likewife tend to place the fubject in a new light.

"I had not long practifed the vaccine inoculation at the hofpital, before I was requefted to extend it into private families in the metropolis, where I foon difcovered that the Cow-pox uniformly appeared in its mildeft form, and was never attended with eruptions. I alfo fupplied feveral medical gentlemen with the vaccine matter, which was ufed by them with the like refult. Hence I began to fufpect that there exifted fome *peculiar* caufe, which rendered the patients under the vaccine inoculation in the hofpital more liable to puftules than others: and that this fufpicion was well founded I have fince, from daily experience, been fully convinced.

"At various times I procured the vaccine virus, as produced in different cows, and with it inoculated patients in the hofpital; but the effects of all the matter I tried were perfectly fimilar: and puftules proved to be not lefs frequently the confequence of thefe trials than of thofe made with the matter formerly employed.

"The laft matter of the vaccine poifon which I introduced into the hofpital, was obtained from Dr. Jenner, and originally taken from Clark's cow, before noticed: with this matter I inoculated at the hofpital on the fame day three patieents, on one of whom about 100 variolous-like puftules were produced. This inftance, and numerous others of the like kind which I could adduce, decidedly prove, that where there can be no doubt entertained of the purity of the Cow-pock matter, with which the patients in the hofpital are inoculated, puftules will frequently be the confequence.

"On the other hand I have to obferve, from daily experience during the laft twelve months, that among the great numbers of children refiding in various parts of London, to whom I have transferred the Cow-pock infection, no inftance of puftules that maturated has occurred. Now, as thefe different effects of the difeafe between the patients in, and thofe out of the hofpital did not depend upon any difference or alteration of the matter with which the inoculations were performed, the only caufe remaining to which the frequent occurrence of puftules on the former can be rationally referred, is the variolated atmofphere of the hofpital, which thofe patients were neceffarily obliged to infpire during the progrefs of the Cow-pox infection.

"Did it not lead me too much into detail, I fhould fhow from many circumftances relating to the patients in the Inoculation Hofpital, that other reafons might from thence be adduced to fupport the opinion here advanced.

"Mr. Evans, Surgeon, at Ketley, in Shropfhire, is the only perfon, except myfelf, who has given an account of the variolous and vaccine inoculations carried on feparately in different perfons at the fame time, and in the fame houfe, fo that feveral of his patients, while under the vaccine infection, were expofed to the variolous effluvia. The number of thofe which he inoculated for the

Cow-pox

Cow-pox amounted to ſixty-eight; and it is worthy of remark, that more than one-half of theſe patients had puſtules. It is true that the eruptions very rarely maturated; but ſtill their frequent occurrence would ſeem to ſhow they aroſe from the ſame cauſe as thoſe at the hoſpital. I ſuſpect alſo, that in thoſe places where the Small-pox is epidemic, or very generally prevailing, the Cow-pox will be found to be equally liable to excite puſtules as in the hoſpital.

"During the very general and fatal prevalence of the Small-pox at a village eight miles diſtant from London, more than 100 perſons were inoculated under my direction for the Cow-pox, of whom one in five had eruptions; and as theſe furniſh the only inſtances which I have experienced, out of the hoſpital, of the Cow-pox producing the variolous-like puſtules, I am diſpoſed to attribute them to the adventitious co-operation of the variolous atmoſphere to which the patients were expoſed. In what way the variolous miaſms act in thus modifying the Cow-pox, or why they co-operate in ſome and not in all caſes of vaccine infection, I ſhall not even venture a conjecture: the cauſes probably will continue as inexplicable as thoſe conſtitutional peculiarities which produce all the varieties of Small-pox."

The practical importance of the following facts, will be a ſufficient apology for our inſerting them.

"In order to ſhow that thoſe who had undergone the Cow-pox reſiſted the infection of the Small-pox, I obſerved in my *Reports*, that upwards of 400 of the patients who had received the former diſeaſe, were afterwards inoculated for the latter, which in no inſtance was produced; though more than 100 of the patients had the vaccine diſeaſe ſo very ſlightly, that it neither produced any perceptible indiſpoſition nor puſtules. In addition to this, I can now ſay, that more than 1000 of thoſe who had undergone the new inoculation, have been put to the ſame teſt, and that the like reſult has been experienced.

"The above facts, added to a multiplicity of others of a ſimilar import, publiſhed by ſeveral profeſſional men, clearly demonſtrate, that the Cow-pox inoculation promiſes moſt important benefits to ſociety; and under this conviction I congratulate the public on the great progreſs it is making, by which the real value of the invention will ſoon become generally acknowledged, and duly appreciated.

"Thoſe who have had much experience in inoculating with the matter of the vaccine pock, muſt have obſerved that it is more apt to fail in communicating the infection than variolous matter, eſpecially if it be ſuffered to dry upon the lancet before it is uſed. This does not ſeem to depend upon the virus of the former being more volatile and more eaſily carried off by evaporation than that of the latter, but from its becoming more hard and leſs diſſoluble upon exſiccation. Care ſhould therefore be taken to moiſten it a conſiderable time before it is uſed. When fluid matter is employed, the lancet ſhould be held nearly at a right angle with the ſkin, in order that the infectious fluid may gravitate to the point of the inftrument, which in this direction fhould be made to fcratch the cuticle repeatedly, until it reach the true fkin, and become tinged with blood. This method has many advantages over the common puncture, and I have found it a more convenient and effectual mode of performing the inoculation than any other. It may be remarked, however, that there are perfons who have never had the Small-pox, and are incapable of receiving it by inoculation, or by any other means whatever. The proportion of thefe to thofe liable to the difeafe has been differently ftated by authors; I have not found them to be more than about one in fixty; but as fuch perfons alfo refift the infection of the Cow-pox, the inoculation of the latter muft therefore fometimes fail, independently of the mode in which it is performed, or of the matter employed."

---

*Reflections on the Cow-pox, illustrated by Cases to prove it an absolute Security against the Small-pox; addressed to the Public, in a Letter to Dr. Jenner, from William Fermor, Esq.* 8vo. pp. 47. London. Robfon and Robinfon.

THE firft part of this Letter contains general obfervations on the Small-pox, and the preference due to the Cow-pox, the marks by which the genuine Cow-pox is diftinguifhed, and a ftatement of the opinions of others refpecting its power of permanently preventing the variolous infection.

The author then obferves, "That the genuine Cow-pox is a certain prefervative againft the Small-pox, I flatter myfelf, the following cafes will fufficiently prove. They are felected from many I could bring, of perfons who having previoufly had the Cow-pox, have never been able to receive the infection of the virus from the Small-pox, though inferted a confiderable time after, and at different periods. Thefe cafes are well known to practitioners and inoculators in this neighbourhood, and I have received moft of them from the parties themfelves.

"Firft Cafe. Jeoffry Tredwell is a reputable farmer, and a tenant of mine, at Chefterton, in this neighbourhood. His brother, William Tredwell, being employed conftantly in milking the cows, was infected with the Cow-pox, and had the difeafe feverely in his hands and fingers. Jeoffry not being engaged fo much in milking as his brother, did not receive the infection. About three years after, thefe two brothers were inoculated with variolous matter, by Mr. Lifter, of Charlbury, an eminent practitioner, at a houfe appropriated for that purpofe. William Tredwell, who had undergone the Cow-pox, could not receive the infection, though he was inoculated feveral times, and remained in the houfe with the other patients. Jeoffry, who had not been infected with it, had a very full Small-pox eruption.

"Second Cafe. Alban Collingridge had the Cow-pox about five or fix and twenty years ago, at his father's farm, at Poodle, which affected his fingers in a violent degree. About four years after, he was three times inoculated for the Small-pox, by Mr. Lifter, without out effect. Two of his brothers, who had never had the Cow-pox, received the variolous infection. He slept with them in order to take it, but no consequence ensued. He has frequently since been exposed to its contagion, and has very lately inoculated his children with the Small-pox, without being in any shape infected with it himself.

"Third Case. Mr. Stevens, a reputable farmer of East Claydon, in the county of Bucks, had the Cow-pox on his farm, in the year 1764.—He himself was infected with it by milking the cows. About four years after, he was inoculated with variolous matter, but without effect. About the year 1791, his family were inoculated with the rest of the parish for the Small-pox, with which they were all infected, but he was not, though he attended them the whole time. This case must appear decisive with regard to the security the Cow-pox matter affords against the variolous infection, as there was a space of twenty-seven years between his having received the disorder from his cows, and his attending his family in the inoculated Small-pox; and an interval of four years between the time he had the Cow-pox, and his being himself inoculated with the Small-pox without effect."

Many other cases are given, equally conclusive with the above; and a list of 326 persons who were inoculated for the Cow-pox, of whom 173 were afterwards inoculated with Small-pox, without receiving the infection in any instance.

Mr. F. concludes his Letter with the following recapitulation: "It is unnecessary for me to say any more on this truly interesting subject. I leave the impartial reader to his natural reflections; but I think, in consequence of the above premises, I may venture to say, that he will now be of opinion that the genuine Cow-pox is mild in its effects, congenial with every situation and employment of life, totally void of contagion, and a certain preservative against the baneful influence of the Small-pox. That no constitutional or family complaint can interfere with its effects, or prevent its adoption.

"To conclude; though the public have certainly great obligations to many distinguished modern practitioners, for having, by the cool regimen and present mode of treatment, considerably abated the natural virulence of the Small-pox, to you alone we are certainly indebted for its complete annihilation."

---

*Medical Jurisprudence. On Madness*; by JOHN JOHNSTONE, M.D. 8vo. pp. 56. London, 1800. Johnson.

THE Author informs us, that this is a part of an extensive work on the subject of medical jurisprudence, and which he should not have published separately, had he not been induced by some recent events. "I have not aimed," says he, "at collecting much of what has been said on the subject of insanity by others. The Anatomy of Melancholy, the works of Battie, Arnold, and Crichton, have exhausted all that can be quoted on the subject, and with Haslam's Practical Inquiry, and the Philosophy of Zoonomia, nearly ly the whole that has ever been faid. To thefe writings therefore the inquifitive reader may refer. I have made an effort indeed to comprefs, not to expand my materials; and fuch as they are, I now deliver them to the public, with the hope that they will tend to familiarife the fcientific doctrines of infanity."

The Author thus fums up his opinions and advice on the fubject of Infanity. "Thus then it appears, that melancholy, lunacy, infanity, madnefs, are the fame difeafe—a difeafe of the organs of the mind, often called into action by vehement paffions, or by injuries of different organs of the body.

"Madnefs can only be deemed an hereditary difeafe, infomuch, as children have a ftructure fimilar to that of their parents who have been mad, and as this peculiar organization is likely to be acted upon by the peculiar manners and habits of the parent in education.

"Madnefs has no lucid intervals; a man is either infane or not infane at a particular moment: unlefs indeed we be allowed to term every period, in which the hallucination of the maniac does not appear, a lucid interval. But this would be moft abfurd, for madnefs is a difeafe of the brain, and fenforial powers, and feldom difcovers itfelf equally at all times. Madnefs is not always diftinguifhable from manner—for it affumes the form of the character, whatfoever that may be.

"The countenance of maniacs is marked by a peculiar wild ftare, not to be miftaken by experienced perfons, generally mixed with a fufpicious or timid, and fometimes with a furious look. Their health is not always vifibly affected, though, for the moft part, the fibres of maniacs, or their powers of motion, are lefs irritable or mobile than in good health; hence they are coftive, and difficult to be purged or vomited. Their fenforial powers, in fome meafure, benumbed; hence they feel pain with lefs acutenefs, and are capable of bearing great extremes of heat and cold, hunger and thirft. Their pulfe is generally flower than common, when there is no irritation nor diforganization.

When it is determined that a man is mad, he ought to be fuppofed incapable of acting. He may perchance act wifely, but reafon being abfent, it is folely from accident if he does fo. A maniac cannot commit crimes, and therefore he ought not to be amenable to human law for their commiffion. He does not difcriminate right from wrong.

"All maniacs fhould be controuled, but all do not require confinement. The neceffity of confinement muft be determined by the degree of fury, by the temper, and the habits of the maniac.

"Finally, Maniacs fhould never be entrufted with the management either of themfelves, or any other perfons, efpecially the young. There is no faculty more familiar to us than that of imitation; it is the firft exercifed by the infant, and it grows with his growth. Maniacs therefore fhould not be fuffered to affociate with young perfons, who will be likely to imitate their actions. For by reiterated imitation, by flow yet certain fteps, we acquire habits,

 which which not only fix the moral character of man, but frequently produce the most pernicious and incorrigible diseases, both of body and mind."

---

*The Edinburgh Practice of Physic and Surgery; preceded by an Abstract of the Theory of Medicine, and the Nosology of Dr. Cullen; and including upwards of Five Hundred authentic Formulæ, from the Books of St. Bartholomew's, St. George's, St. Thomas's, Guy's, and other Hospitals in London, and from the Lectures and Writings of the most eminent public Teachers. With four Quarto Plates, neatly engraved, representing the different Instruments used in Surgery.* 8vo. pp. 920. price 14s. in boards. London, 1800. Kearsley.

This comprehensive Compilation may be considered as a Medical Library for young students; and we think it may, for some time, supercede the necessity of any other. It will serve the purpose of a Text-book during their attendance on Medical and Surgical Lectures, and we may add Midwifery; for the diseases of pregnancy, the puerperal state, and those peculiar to infants, are contained in it. We also find several diseases not treated of by systematic writers in general, but only in distinct works, as Angina Pectoris, Poisons, Suspended Animation, Cow-pox, &c. The work is furnished with Indices to the Practice of Physic, the Surgery, the Materia Medica, and the doses of medicines, as well as of the new and old names.

We recommend this book as a valuable addition to the library of the apothecary's shop.

---

*Nosology: or, A Systematic Arrangement of Diseases, by Classes, Orders, Genera, and Species; with the distinguishing Characters of each, and Outlines of the Systems of Sauvages, Linnæus, Vogel, Sagar, and Macbride. Translated from the Latin of W. Cullen, M. D. late Professor of the Practice of Physic in the University of Edinburgh.* 8vo. pp. 238. London, 1800. Robinsons.

This Translation appears to us to be executed with great accuracy and fidelity; and is accompanied with the necessary Indices, and a Table of the Greek derivations of the names of the classes, orders, and genera.

---

*A Practical Inquiry into Disordered Respiration; distinguishing the Species of Convulsive Asthma, their Causes, and Indications of Cure. The second Edition, corrected, with an Appendix.* By Robert Bree, M. D. 8vo. pp. 300. London, 1800. Robinsons.

The design of this industrious Author is to ascertain the real causes of Asthma, and thence the species and method of treatment. The first edition, he informs us, "was published with some precipitation, and had the faults of diffuseness, and want of perspicuity; but its defects did not prevent the sanction of it in this country, nor on the continent, where it has been translated into two languages."

After a comprehensive view of the opinions of others respecting the *ratio symptomatum*, and stating his objections to the doctrine of spasmodic

ſpaſmodic conſtriction, Dr. B. at p. 88, proceeds to the *diagnosis*, which we ſhall tranſcribe as a ſpecimen of the reaſoning and ſtile.

" Aſthma is ſo ſtrongly marked, that there can be little difficulty in knowing the diſeaſe; yet it will elucidate the ſubject, to ſhow its diſtinction from ſome other diſeaſes to which it may bear an analogy in its cauſes and effects.

" Defluxions on the upper part of the lungs and Sneiderian membrane are uſually inflammatory affections of the mucous glands which line the paſſages of the noſe, fauces, and trachea, as far as its diviſions, and poſſibly lower in the breaſt, but not extending to the extremities of the air pipes.

" The catarrhal diſpoſition is very frequently followed by Aſthma, becauſe repeated inflammation of the capillary veſſels and mucous excretories, may induce, in ſome habits, a loſs of tone, which may prevent their reſiſtance to a circulatory impulſe even leſs than healthy, and ſubject them to the influence of exciting cauſes of little force in compariſon with what they formerly ſubmitted to. For this reaſon, elderly perſons have their natural excretion of mucus much more copious, as they may have been more affected by catarrh, and they are accordingly more liable to Aſthma. If inflammatory diſpoſition be not wholly loſt in theſe perſons, by the progreſſive debility of the veſſels of the lungs, Peripneumonia Notha will be the character of the pulmonary diſeaſe, attended often with great danger.

" I am aware that this ſpecies will be deſignedly confounded by many reaſoners with the Humoral Aſthma; but it is time that the diſtinction of *Humoral* and *Convulſive* ſhould be better underſtood. If mucus be diſcharged in greater quantity in one caſe than in another, the reſpiratory actions being the ſame in both, there is no good reaſon for calling one only Convulſive. Is not every Humoral Aſthma Convulſive? If the unfortunate patient have ſo little irritability as not to be excited to cough and expectorate, the phlegm muſt ſuffocate him, if the abſorbing veſſels do not carry it off: and this proceſs is attended by Convulſive reſpiration. But though the Humoral muſt be Convulſive, the Convulſive Aſthma is not always Humoral; for we ſhall ſee that irritation may exiſt in a more ſubtle form than lymph.

" It is conſiſtent with the rules of the animal œconomy, th t Catarrh ſhould not be indicated by thoſe violent contractions of the muſcles of reſpiration which take place in Convulſive Aſthma. Fever attends both Catarrh and Phthiſis; and we may obſerve, though we cannot aſſign a reaſon for the fact, that if fever ſuperſedes, it generally terminates convulſive motions.

" If Catarrh occaſionally lead to Aſthma, it ſtill oftener brings on *Phthiſis*, a diſeaſe which depends on a ſtate of the lungs, oppoſite to that which permits ſerous effuſion.

" In Aſthma, an exceſs of blood in the pulmonary veſſels may very probably precede the exhalation of the finer part into the veſiculæ and bronchia; this plethora is local, ariſing from the relaxed texture of the coats of the veſſels, and relieves itſelf by effuſion.

" In incipient *Phthiſis* the arterial impulſe is more conſiderable than in health, but the pre-diſpoſition of the pulmonary veſſels is not favourable to a relief by effuſion, till the fever has acquired ſtrength, and coagulable lymph inſtead of pelucid ſerum comes to be effuſed.

" It is therefore to be allowed, that there is a prediſpoſition, in conſequence of which inflammation will affect the arterial extremities, and produce Phthiſis, as doubtleſs there is a pre-diſpoſition leading to that atonic ſtate of the vaſcular pulmonary ſyſtem producing Aſthma.

" There may be alſo an intermediate ſtate, in which a balance is preſerved between the criſis of inflammation ſealing up the orifices of the arterial exhalents, and their diſtenſion ſo gradually acquired as to permit the escape of the finer fluid, and consequent relief of arterial fulness; but it is probable that this balance cannot be long adjusted where the pre-disposing causes have had a considerable influence, and that if the exhalents do not dilate soon in consumptive habits, Phthisis must take place; and in persons of an opposite constitution, which I conceive is favourable to Asthma, the effusion of lymph into the vesiculæ and bronchia will determine, in no long time, the future character of the pulmonary disease.

" *Lethargic affections* have been considerably allied to pulmonary complaints in the consideration of many authors; so much so, as to create a question if the cause of lethargy did not exist in the lungs.

Hippocrates says, " lethargic diseases are the same as pneumonic, and not " altogether different from the humid peripneumony." (Peripneumonia Notha.) Some of his commentators have even defended this opinion by the practical remark, that lethargy is critically relieved by expectoration of purulent or serous fluid.

" The lethargic symptoms in Asthma and Peripneumony are sufficiently accounted for by the interruption to the course of the blood from the right side of the heart to the left, obstructing the influx of venous blood from the head. The natural consequence in bad cases of Asthma is Apoplexy.

" Asthma being thus occasioned by serum in the vesiculæ, may be considered as an *Hydropic Disease*; but it is obviously distinct from Hydrothorax, in which the water is collected in the sacs of the pleura, or cellular texture of the lungs. In each situation it will occasion dyspnœa, which, though subject to exacerbations, from accidental causes, will not put on the form of Periodic Asthma.

" We have sufficient testimony of the connection of Asthma with Dropsy in the histories of practical authors, frequently pointing out the intercurrence of symptoms and changes of one disease into the other, when Asthma has been of long standing: some proof of this is contained in Sect. VI. of this Inquiry.

" Hoffman and Willis have particularly noticed the hydropic appearances of the feet, and the tendency to general Dropsy in Asthma: and the observation of these authors is supported by that of other practitioners.

" Sydenham opens his treatise of the Dropsy, by stating the first symptom of that disorder, to be the swelling of the legs, and pitting of the ancle by pressure of the finger: but this is not so certain a sign in women as in men; nor even in the latter is it to be considered as an absolute certainty of the disease having commenced. He then proceeds with the following observation:

" Etenim cum senex quispiam, habitu corporis paulo pleniori præditus, " *Asthmate* jam a multis annis laborans, ab eodem derepente, idque hye- " mis tempore, fuerit liberatus, mox ingens tumor musculos tibiarum oc- " cupabit, hydropicorum tumores æmulans, qui hyeme etiam magis quam " æstate, tempestate magis pluvia quam serena, pariter invalescit, et tamen " sine quovis incommodo insigniori, eundem ad libitinam usque comitabi- " tur. Quo non obstante, si generaliter loquamur, suræ et tibiæ intumes- " centes, etiam in viris, pro signo supervenientis hydropis habendæ sunt; " maxime si ita affecti spiritum ægrius ducant."

" This sagacious observer might have attributed the swelling of the legs, with great truth, to hydropic effusion in the Asthmatic, as well as in other cases; and the cessation of the Asthma when these swellings commenced, seems to corroborate, beyond dispute, the theory, that both affections depended on one cause. The swelling was larger in winter than in summer, in moist weather than in dry. Alterations in the atmosphere rapidly affect the Asthmatic, and change his habit from a perspiring to an imbibing state, and whether the aqueous collection stagnate in the vesicles of the lungs, or be be taken up by the abforbents, and be again effufed in the lower extremities, the identity of the caufe is fufficiently plain.

"Infanity fometimes fufpends Confumption, and Confumption Infanity: Afthma, likewife, is fucceeded occafionally by Infanity; probably from the turgid ftate of the veffels of the head in confequence of the difficulty with which the right fide of the heart propels the blood through the lungs to the left. In the hydropic diathefis, fo frequently accompanying advanced Afthma, the difeafe of the head is ftill more frequent.

"The following two cafes deferve attention. In one, the patient had alternately Afthma and Infanity: in the other, there appeared Anafarca and Infanity at the fame time. The treatment was fuccefsful, though founded on the fole indication of curing the Dropfy.

"We conclude then, that Afthma, Infanity, and Dropfy, had the fame caufe; for, if Infanity and Afthma were one difeafe, and Infanity and Dropfy were one difeafe, *Afthma* and *Dropfy* muft be one difeafe.

"From a confideration of the caufes of Infanity by the learned Dr. Arnold, there can be no difficulty in affenting to the connection between thefe difeafes."

The Author next proceeds to the inveftigation of the predifpofing and exciting caufes, and the eftablifhing of his *four* fpecies. He then lays down a plan of treatment for the paroxyfm of each fpecies, with general rules for the *diet* of the patients, both during the paroxyfms and the intermiffions.

The Appendix contains a recapitulation or fynopfis of the work, with additional facts and obfervations in confirmation of his diftinctions, and plan of treatment.

Though the ftile of this work appears to us defective, we have no hefitation in recommending the matter of it to practitioners, as well worthy of their attention.

---

*Refearches, Chemical and Philofophical; chiefly concerning Nitrous Oxyde, or Dephlogifticated Nitrous Air, and its Refpiration. By* HUMPHRY DAVY, Superintendent of the Medical Pneumatic Inftitution. 8vo. pp. 590. London, 1800. Johnfon.

THE firft of thefe Refearches chiefly relates to the production of nitrous oxyde, and the analyfis of nitrous gas and nitrous acid. "In this," fays the Author, "there is little that can be properly called mine; and if by repeating the experiments of other chemifts, I have fometimes been able to make more minute obfervations cconcerning phænomena, and to draw different conclufions, it is wholly owing to the ufe I have made of the inftruments of inveftigation difcovered by the illuftrious fathers of chemical philofophy, Cavendifh, Prieftley, Black, Lavoifier, Scheele, Kirwan, Guyton, Berthollet, &c. and fo fuccefsfully applied by them to the difcovery of truth.

"In the fecond Refearch, the combinations and compofition of nitrous oxyde are inveftigated, and an account given of its decompofition by moft of the combuftible bodies.

The third Refearch contains obfervations on the action of nitrous oxyde upon animals, and an inveftigation of the changes effected in it by refpiration.

In the fourth Refearch the hiftory of the refpirability and extraordinary ordinary effects of nitrous oxyde is given, with details of experiments on its powers made by different individuals."

Our recommendation will not be necessary to induce every lover of chemistry and physiology to peruse this truly philosophical work; our readers will judge of its importance by the following extracts.

Mr. D. concludes his first Research with the following general remarks on the production of nitrous oxyde. " There are no reasons for supposing that nitrous oxyde is formed in any of the processes of Nature; and the nice equilibrium of affinity by which it is constituted, forbids us to hope for the power of composing it from its simple principles. We must be content to produce it, either directly or indirectly, from the decomposition of nitric acid. And as in the decomposition of nitrate of ammoniac, not only all the nitrogene of the nitric acid enters into the composition of the nitrous oxyde produced, but likewise that of the ammoniac, this process is by far the cheapest, as well as the most expeditious. A mode of producing ammoniac at little expence, has been proposed by Mr. Watt. Condensed in the sulphuric acid, it can be easily made to combine with nitric acid, from the decomposition of nitre by double affinity. And thus, if the hopes which the experiments at the end of those researches induce us to indulge, do not prove fallacious, a substance which has been heretofore almost exclusively appropriated to the destruction of mankind, may become, in the hands of philosophy, a means of producing health and pleasurable sensation."

Respecting the decomposition of nitrous oxyde and its analysis, he draws these general conclusions. " From what has been said in the preceding sections, it appears that the inflammable bodies, in general, require for their combustion in nitrous oxyde, much higher temperatures than those at which they burn in atmospheric air, or oxygene.

" When intensely heated they decompose it, with the production of much heat and light, and become oxygenated.

" During the combustion of solid or fluid bodies, producing flame, in nitrous oxyde, nitrous acid is generated, most probably from a new arrangement of principles, analogous to those observed in Sect. II. by the ignition of that part of the gas not in contact with the burning substance. Likewise when nitrous oxyde in excess is decomposed by inflammable gas, nitrous acid, and sometimes a gas analogous to common air, is produced, doubtless from the same cause.

" Pyrophorus is the only body that inflames in nitrous oxyde, below the temperature of ignition.

" Phosphorus burns in it with the blue flame, probably forming with its oxygene only phosphoreous acid at the dull red heat, and with the intensely vivid flame, producing phosphoric acid at the white heat.

" Hydrogene, charcoal, sulphur, iron, and the compound inflammable bodies, decompose it only at heats equal to, or above, that of ignition: *probably*, each a different temperature.

" From

"From the phænomena in Sect. V. it appears, that at the temperature of intense ignition, phosphorus has a stronger affinity for the oxygene of nitrous oxyde than hydrogene; and reasoning from the different degrees of combustibility of the inflammable bodies, in mixtures of nitrous oxyde and nitrogene, and from other phænomena, we may conclude with probability, that at about the white heat, the affinity of the combustible bodies for oxygene takes place in the following order: Phosphorus, hydrogene, charcoal, iron, sulphur, &c.

"This order of attraction is very different from that obtaining at the red heat; in which temperature charcoal and iron have a much stronger affinity for oxygene than either phosphorus or hydrogene.

"The smallest quantity of oxygene given in the different analyses of nitrous oxyde just detailed, is thirty-five hundred parts; the greatest proportion is thirty-nine.

"Taking the mean estimations from the most accurate experiments, we may conclude that 100 grains of the known ponderable matter of nitrous oxyde, consist of about 36,7 oxygene, and 63,3 nitrogene; or, taking away decimals, of 37 oxygene to 63 nitrogene; which is identical with the estimation given in Research I.

"Analysis and synthesis clearly prove that oxygene and nitrogene constitute the known ponderable matter of atmospheric air, nitrous oxyde, nitrous gas, and nitric acid.

"That the oxygene and nitrogene of atmospheric air exist in chemical union, appears almost demonstrable from the following evidences.

"1st. The equable diffusion of oxygene and nitrogene through every part of the atmosphere, which can hardly be supposed to depend on any other cause than an affinity between these principles.

"2dly. The difference between the specific gravity of atmospheric air, and a mixture of 27 parts oxygene and 73 nitrogene, as found by calculation; a difference apparently owing to expansion in consequence of combination.

"3dly. The conversion of nitrous oxyde into nitrous acid, and a gas analogous to common air, by ignition.

"4thly. The solubility of atmospheric air un-decompounded in water.

"Atmospheric Air, then, may be considered as the least intimate of the combinations of nitrogene and oxygene.

"It is an elastic fluid, permanent at all known temperatures, consisting of ,73 nitrogene, and ,27 oxygene. It is decomposable at certain temperatures, by most of the bodies possessing affinity for oxygene. It is soluble in about thirty times its bulk of water, and as far as we are acquainted with its affinities, incapable of combining with most of the simple and compound substances. 100 cubic inches of it weigh about 31 grains at 55° temperature, and 30 atmospheric pressure.

"Nitrous Oxyde is a gas unalterable in its constitution, at temperatures below ignition. It is composed of oxygene and nitrogene, trogene, exifting *perhaps* in the moft intimate union which thofe fubftances are capable of affuming. Its properties approach to thofe of acids. It is decompofable by the combuftible bodies at very high temperatures, is foluble in double its volume of water, and in half its bulk of moft of the inflammable fluids. It is combinable with the alkalies, and capable of forming with them peculiar falts. 100 grains of it are compofed of about 63 nitrogene, and 37 oxygene. 100 cubic inches of it weigh 50 grains, at 55° temperature, and 30 atmofpheric preffure.

"NITROUS GAS is compofed of about 56 oxygene, and 44 nitrogene, in intimate union. It is foluble in twelve times its bulk of water, and is combinable with the acids, and certain metallic folutions; it is poffeffed of no acid properties, and is decompofable by moft of the bodies that attract oxygene ftrongly, at high temperatures. 100 cubic inches of it weigh about 34 grains, at the mean temperature and preffure.

"NITRIC ACID is a fubftance permanently aëriform at common temperatures, compofed of about 1 nitrogene, to 2,3 oxygene. It is foluble to a great extent in water, and combinable with the alkalies, and nitrous gas. It is decompofable by moft of the combuftible bodies, at certain temperatures. 100 cubic inches of it weigh, at the mean temperature and preffure, nearly 76 grains."

The third Refearch contains a great number of experiments on the changes produced in them, by the refpiration of nitrous oxyde, and other gaffes; and concludes thus:

"The experiments in the firft Divifion of this Refearch, prove that nitrous oxydes when refpired by animals, produce, peculiar changes in their blood and in their organs, firft connected with increafed living action; but terminating in death.

"From the experiments in this Divifion, it appears, that nitrous oxyde is rapidly abforbed by the circulating venous blood, and of courfe its condenfed oxygene and nitrogene diftributed in the blood over the whole of the fyftem.

"Concerning the changes effected in the principles of the impregnated blood during circulation and its action upon the nervous and mufcular fibre; it is ufelefs to reafon in the prefent ftate of our knowledge.

"It would be eafy to form theories referring the action of blood impregnated with nitrous oxyde, to its power of fupplying the nervous and mufcular fibre with fuch proportions of condenfed nitrogene, oxygene and light, or etherial fluid, as enabled them more rapidly to pafs through thofe changes which conftitute their life: but fuch theories would be only collections of terms derived from known phænomena, and applied by loofe analogies of language to unknown things.

"We are unacquainted with the compofition of dead organifed matter; and new inftruments of experiment and new modes of refearch muft be found, before we can afcertain even our capabilities of difcovering the laws of life."

The

The last Research, which is by far the most important to the practitioner, details the effects produced on the human frame, by the respiration of nitrous oxyde and other gases. For these we must, at present, refer to the Work itself.

---

*The Doctrine of Phlogiston established, and that of the Composition of Water refuted: by* JOSEPH PRIESTLEY, LL.D. F.R.S. &c. &c. 8vo. pp. 90, 1800. London, Johnson.

It is inconsistent with our plan to enter into theoretical discussions which are not immediately connected with the healing art. Whether the truth lie on the side of Dr. P. or his opponents, we cannot determine; but we believe that the tide runs so forcibly at present in favour of the Antiphlogistic doctrines, that chemists had rather be carried away by it, than be at the trouble of stemming the current, even for the sake of arriving at the harbour of truth.

Dr. P. examines the calcination and reduction of metals, the decomposition of water, the constitution of fixed air, and azote, from all which he draws inferences in favour of the existence of Phlogiston.

---

*A Practical Treatise on the Efficacy of Stizolobium, or Cowhage, (the Dolichos Pruriens of Linnæus) internally administered, in Diseases occasioned by Worms. To which are added, Observations on other Anthelmintics of the West Indies. By* WILLIAM CHAMBERLAINE, Surgeon, Fellow of the Medical Society of London, 8vo. pp. 90. London, Printed for the Author, No. 29, Aylesbury Street, Clerkenwell.

We think this practical work well worthy the attention of practitioners: It contains a sufficient number of cases to prove the efficacy of the remedy, as well as the safety with which it may be administered.

---

*Critical Survey of the latest Theories on difficult Dentition in Children.*

[ Continued from pp. 171—177 of our last. ]

Notwithstanding these authorities, it will appear by the following observations, how little it deserves to be depended upon, and how far it is from being capable of removing those affections, which befall the infantile age in the period of teething.

1. It seems very improbable, and almost ridiculous to imagine, that, by this simple operation, the growth of the teeth should be promoted in a few days, to which otherwise several months are required.

2. It has been stated, that convulsions have actually ceased after performing that operation; but could not its effect be thus explained, that they were removed by its having caused an irritation in another part of the body, and acted in a similar manner as we observe blisters, scarifications, and artificial ulcers, applied for the same purpose?

3. As it is frequently the cafe, that more teeth than one are preparing to penetrate, it is difficult to determine which protuberance is to be cut through.

4. The fuccefs of the operation depends in a great meafure on the critical time when it fhould be undertaken, which is often impoffible to determine. Van Swieten advifes, therefore, not to perform it for fear of lofing credit, as he relates a cafe where the tooth did not appear till two months after the operation.

5. By the experience of eminent phyficians and furgeons, it has been found not only of no avail, but rather hurtful in fome cafes. Platner (Inftitutiones Chirurg. § 1078) advifes not to undertake the operation, for fear of hurting the teeth. Schaeffer obferves, in his tranflation of Armftrong, that only in a few cafes it feemed to have effect, but in two others it proved unfortunate. Deffault and Gavand, (Traité d'Ofteologie) confefs, that it has been performed without fuccefs. Dr. Selle and Dr. Tade, two phyficians of known merit, caution every body againft it, becaufe they think that the wound, by healing and caufing a fcar, will render the piercing of the teeth more difficult. Dr. Starke, Profeffor and an eminent practitioner at Jena, and Doctor Hecker, are likewife oppofers of the operation.

The application of leeches behind the ear is another remedy, whofe infallibility in difficult dentition was highly praifed by Le Roy, (in Efprit des Journaux, 1784, where he fays of it, " C'eft donc un grand moyen de population, qu'une fangfue derniere l'oreille des enfans") though it was recommended before him by Harris, (de morbis infantum); but it has been now much left off, and only kept its reputation againft difficult dentition when another difeafe was miftaken for it and accidentally cured. The fame may be faid of other general remedies, which are likewife efficacious in other difeafes, but which poffefs no particular power againft difficult dentition, as blifters, &c.

Having given the arguments againft difficult dentition, it may be proper to add fome general reflections on the fubject.

1. If any danger could be fuppofed to arife to children from the growth of bones, and if Nature did not prevent any morbid affections, or furmount any difficulties by proceeding always gradually in the formation of the body, dangerous fymptoms might be much fooner expected from the growth of the fkull, particularly when the futures are forming, becaufe the dura mater and the pericranium are more fenfible than the foft fpungy gingivæ; and even the growth of the nails might more eafily be fuppofed the caufe of thofe fymptoms, on account of the great fenfibility of the neighbouring parts: but nobody ever confiders them as caufes of morbid infantile affections.

2. There is nothing analogous in the economy of the human body, or which has any fimilarity with difficult dentition, wherein by a gradual and natural growth difeafe is produced. The growth of other bones would as certainly excite pains and dangerous fymptoms, if it was fudden or very quick; which being anomalous, lous, is never ſuppoſed to be the caſe; and pains are not even perceived in ſome diſeaſes of the bones, in curvatures of the back-bone, or when teeth grow in a preternatural direction.

3. If the growth of teeth were able to cauſe ſuch very dangerous ſymptoms, it may be ſuppoſed, that triſmus would be particularly brought on, as it is ſo common to children; but this is only obſerved in the firſt days of life.

4. Pains ariſing from double teeth cutting through in adults, are not to be compared with dentition in children, becauſe this is preternatural and anomalous. It is with more difficulty ſuch a tooth can get through, becauſe the place which it is to occupy, is more contracted, and partly filled with the neighbouring teeth, and beſides this, the gums are not ſo ſoft and yielding as they are in a child.

5. There is no analogy between the pains ſuppoſed to be occaſioned by difficult dentition and thoſe ariſing from a caries dentium, becauſe a true inflammation ſpreading to the neighbouring parts takes place here, which originates from the nerve of the teeth being affected. Carious teeth never produce ſuch general ſymptoms, even in children, as are derived from difficult dentition; every affection remains local here, and only a little fever, or a want of appetite comes on, as is the caſe in every pain. Now is it probable, that a difference of a few years would leſſen the irritability ſo much, that, for inſtance, a child of ſix years ſhould not be attacked with convulſions from a viſible and painful affection of a tooth? whereas, it is ſuppoſed to ſuffer them at the age of one or two years from an inviſible and ſuppoſitious cauſe.

Dr. Wichmann concludes his paper by obſerving, that even if he had not ſucceeded in proving the non-exiſtence of difficult dentition, he ſhould think himſelf ſatisfied if he could but convince practitioners not to attribute too much to dentition; that this was not aſſigned as the only cauſe for all diſeaſes of children, obſervable at the time of dentition; and that it may not be neglected to point out and ſearch other cauſes, before they are derived from dentition.

Such is the opinion of Dr. Wichmann; on which we ſhall ſubjoin, (in our following Number) a few Obſervations, ſtating how far it is to be reſtrained or admitted.

---

*Bemerkungen;* i. e. Remarks on the Brunonian Practice; by D. Christ. William Hufeland, Profeſſor of Medicine at Jena, Vol. I. 1799, pp. 127, 8vo. Tubingen, Lotta.

The name of Profeſſor Hufeland has acquired ſo much celebrity, even in this country, as naturally to excite the curioſity of every medical man, to know the opinion of this great practitioner, on a ſyſtem of medicine which has of late gained ſo much ground amongſt phyſicians, and has been particularly received with applauſe on the continent. Mr. Hufeland had already communicated his judgment on it, in ſeveral numbers of his Practical Journal; but we find his ideas all united here, though, except in a few

 inſtances inftances, unaltered. In *the Introduction,* he confeffes himfelf to be, on the whole, an antagonift of this new fyftem, in which but few ideas meet with his approbation, that, however, have been already received before Brown, by every reafonable practitioner. But, the chief object of this publication is, to caution young practitioners againft indifcriminately applying in practice the raw principles of Brown's theory, feduced by its apparent fimplicity and confiftency. The manner, he proceeds, in which fome enthufiafts attempted to introduce the Brunonian practice, by which they aimed at nothing lefs than a revolution in medicine; the fpirit of faction which is thereby excited, is by no means calculated to forward fcience. It lies indeed, in the nature of things, that the erroneous principles and doctrines of Brown's theory will be fupplanted in time; but they may leave a dangerous tendency to faction and difcord, which is very prejudicial to truth and knowledge, and apt to lead to partiality, which checks the mind in inquiry. This fyftem proceeds but from one point of view, that of incitability, and deems all auxiliary knowledge, anatomy, chemiftry, &c. fuperfluous. *The introduction* concludes, that the Brunonian fyftem is full of inaccuracies and fophiftry, and has too many chafms to deferve the name of a fyftem: Pure Brunonianifm will produce daring phyficians, not underftanding how to manage Nature, but continually tyrannifing over her. Againft thefe introductory premifes, may be replied by the well-wifher of that fyftem, that, if its principles are allowed to be theoretically confiftent and juft, their not proving fo in practice is to be derived from their being mifunderftood and mifapplied, which, confequently, cannot be confidered as a fault of the fyftem, but rather as a fault againft it. Its imperfection can be no objection; this it fhares with all fyftems, by which Nature is regulated. However, it excels all other, by fixing only one point of view, and avoiding a perplexing multiplicity; farther, it may be fuggefted, that ill confequences fuppofed to originate from the Brunonian method of treatment, are partly exaggerated, partly groundlefs, if this is built not upon the mere literal fenfe, but upon its true genius, a circumftance which deferves to be well weighed and diftinguifhed. Incitability is by no means the only point of view in this fyftem; and the extenfive clafs of topical difeafes, leaves great room for mechanic action and chemiftry. The ftudy of the auxiliary branches of medicine, is not at all rendered fuperfluous by it, as the celebrated *Frank,* of Vienna, has fufficiently demonftrated. How far this general apology for the Brunonian fyftem is able to remove the above imputations, we muft leave undecided, as this would neceffarily lead us farther into the fubject than the limits of this Journal will admit. It may fuffice to remark, that many antagonifts of that fyftem, who attempted to fhow its errors and infufficiency, have fometimes hurt their own caufe, by not having fufficiently ftudied and entered its genius. Whether this is the cafe here, we leave out readers to judge, by reprefenting to them briefly, the contents of this publication, to which the bounds of the the Medical Journal will only allow us to add, now and then, fome remarks, which can be brought forward on the fide of the Brunonian theory. The contents accordingly, are delivered under eleven different heads: 1. *Practical point of view.* In the examination of a fyftem, it is not of fo much confequence, whether it poffeffes a logical confiftency, but whether it alleviates and improves the cures of difeafes; and many fyftems have been forfaken, on account of this difharmony with the practice. 2. *Diagnofis of difeafes* is but feemingly rendered eafier by Brown's theory; for the fymptoms of a fthenic ftate often occur in afthenic difeafes, and vice verfa. Brown commits, befides, the fault of confidering the names of difeafes fufficient to diftinguifh whether they are fthenic or afthenic. Moreover, the diftinction of direct and indirect debility, is fubject to many difficulties, and often quite impoffible; for inftance, in fpeechlefs perfons; and fometimes fuch caufes have preceded, that may produce as well one ftate as the other, which muft of courfe perplex the Brunonian phyfician, or oblige him at leaft to ftate a mixed debility, in which exifts at the fame time, want and fuperabundance, a ftate quite contradictory in itfelf, which requires alike the moft powerful and the weakeft ftimuli. Finally, it is very often impoffible to find and determine the degree of incitation, and the proper remedy for it. Every thing depends on a cautious experiment; and the pretended mathematical certainty of the Brunonian theory is but a phantom. To this will be probably objected, that fthenic and afthenic fymptoms are not only attended to in this fyftem, but that, alfo, a chief point of it confifts in determining whether a difeafe is topical or general; and as the prefent phenomena of a fthenic and afthenic ftate are often uncertain and fallible, it is particularly urged by this fyftem, to look back to the preceding influences and morbific difpofition for determining the diagnofis. 3. *The two fpecies of death.* Prof. H. thinks it inconfiftent with experience, that according to this fyftem, there are but two paths to death, by direct and indirect debility: there exifts a third, in the unfitnefs or deftruction of vital organs, where neither the afthenic nor fthenic method is of any avail. However, the Brunonian will refer here to the above proximate caufe, either by confidering the lofs of fuch an organ as the want of a ftimulus neceffary for life, or as a ftate primarily originating from direct or indirect debility, &c. 4. *Action of medicines.* It is a chief principle of the Brunonian theory, that the action of remedies depends folely on gradation, or the degree. However, its followers often combine indifcriminately, remedies of different degrees, viz. bark and fenega. Now, if the degree of incitement requires bark, it ought to be given alone; for fenega being a weaker ftimulus, cannot add to its power, nor diminifh it, as it is itfelf an exciting remedy: the ufe of fuch a combination is, however, proved by experience. In this manner, the Brunonian is obliged to act, inconfiftent with his fyftem, and filently to allow cafes, where a fpecific difference of the ftimuli and their chemical action take place. As inftances of a mere mechanic and chemical action, the ufe of oil in inflammations, of alkali in gout, of of citric acid in fcurvy, is adduced. But the gradual difference of remedies in their action, muft be only underftood as far as they act upon incitability in general, but not on the individual ftate of a fingle organ, where different laws take place. With refpect to the combination of different remedies, it may be faid, that this apparent inconfiftency depends on our imperfect knowledge of the relative and abfolute powers of medicines, and of the prefent ftate of incitation; and accordingly, in cafes where even a cautious experiment is not allowed, recourfe muft be had to a combination of remedies, which experience has found to produce the neceffary degree of incitation. The mechanic ufe of oil in inflammations is by no means denied by Brown's theory, and alkalies might be fuppofed not to act as ftimuli, if they cured nothing but the gout, and if this was only cured by them. The ufe of citric acid, though feemingly a weakening medicine, may, in a high degree of fcurvy, be in the fame way explained, as the ufe of ice in frozen animals. 5. *Unequal diftribution and action of vital power.* Under this title, Mr. H. enters into an enquiry of the fthenic and afthenic method, as a bafis of the Brunonian theory; and attempts to fhow, how repugnant this is to reafon and experience. The different parts of the body poffefs different degrees, and fpecific different modifications for irritaments; as thefe act either only upon one part, or in different degrees upon all, and produce confequently, in one, a fthenic, in another an afthenic ftate. This feems to be confirmed by experience in nervous fevers and pulmonary confumptions; where, in the firft cafe, active inflammations fometimes occur, requiring moderate bleeding and antiphlogiftics, when the general treatment confifts in keeping up the nervous power by valerian, camphor, &c; in the latter, fmall venæfections often contribute to prolong the life of the patient. It may fuffice to fuggeft here, that according to Brown, both ftates are not entirely denied to co-exift in different parts of the fame organifms, and that there is no fpecific irritament, which does not prove for the whole conftitution either afthenic or fthenic. The inftances befides adduced by Mr. H. are doubtful, and not even admitted by fome Non-Brunonians. 6. *Action of cold and warmth.* Prof. H. difcuffes, in a dialogue between himfelf and a friend of the Brunonian fyftem, the queftion, whether cold has a weakening, and warmth a ftrengthening effect upon the body. He propofes feveral arguments to prove, that this expreffion is very relative and unfettled; that warm baths are known to render the pulfe flow; that inflammatory difeafes occur oftener in winter than in fummer; in fhort, that ftrengthening and weakening are ideas too relative, to be able to afcertain whether any thing is weakening or ftrengthening; that cold has, befides, an aftringent quality, &c.; in fhort, the contrary of this Brunonian principle may with the fame reafon be afferted. How far thefe and fimilar arguments may appear fatisfactory to a Brunonian, is eafily conceived. 7. *Application of cold and warmth.* Prof. H. relates here, the cafes and circumftances at large, in which both may be ufed as ftimuli, as ftrengthening and weakening remedies, according according to what we find recorded by experience. Some of these seem indeed contradictory to Brunonian principles, or would at least, with difficulty be explained after them: however, a Brunonian will not be at a loss to extricate himself. 8. *Hæmorrhagies.* There are not only passive or asthenic, but likewise active or sthenic hæmorrhagies, originating from an increased action or local irritation, either idiopathic or consensual, which require weakening remedies, antispasmodics, &c. 9. *Direct and indirect debility.* The notion of two forms of debility from increased or diminished irritabiliy, was entertained long before Brown received and influenced the practice; but it is erroneous to derive them, with him, from the different action of irritaments. In support of this, Prof. H. brings forth several arguments; viz. that the same causes produce quite contrary species of debility; that often a vicissitude of both species of debility occurs in the same individual, without the least change in the external influences; in short, that Brown's distinction of direct and indirect debility has no practical use. 10. *Topical and general diseases.* The division of diseases into topical and general, already adopted by Galen, is by no means calculated to improve the practice, because no certain mode of treatment can be built upon it, as there are topical diseases, the causes of which lie in the general constitution; viz. scorbutic ulcers, &c. as general diseases are often cured by topical remedies, blisters, purgatives, &c. The division into idiopathic and consensual diseases, seems therefore, by far preferable. By this it appears, that Prof. Hufeland has not quite entered into the notion which Brown has of topical diseases, which is different from what has been stated here by him. 11. *Necessary retrospect to the state of the organic as well as morbid matter.* This circumstance seems to be utterly neglected by Brown, by only adopting the notion of incitation and incitement. However, many diseases do not depend on the degree of incitation, but on the chemical difference of organic materia, because, it would be otherwise inconceivable, why debility could produce scurvy as well as gout; why alkali is hurtful in the latter, and useful in the former. Remedies do not therefore merely act as irritaments, but they cause also a change in materia. The whole is concluded with some cases, as additional arguments against the fashionable practice: Such are the objections of Prof. Hufeland. The plan of this Journal will excuse us for not having reviewed them more at large; and we only beg leave to add, that though the above arguments show the ingenuity of their author, yet they will not be deemed sufficient by the Brunonian, to subvert the system against which they are intended.

---

*Apologie, &c.* i. e. Apology for the Brunonian System of Medicine, grounded upon Reason and Experience. Edited by D. Charles Werner, Physician in Austria, Vol. I. pp. 314, 8vo. Vienna, Wappler and Beck.

The tendency of this publication is intimated upon the title, and the author informs us in the Introduction, that it is intended to defend Brown's doctrine, and confirm it by facts; a doctrine which he he pretends to be, notwithſtanding all its excellencies, ill uſed by its antagoniſts. For this end, he invites all thoſe who believe in it, to ſupply him with communications tending to the purpoſe. In the inquiry, however, upon medical ſubjects, it ſhould be with every body a conſtant rule, not to proceed from any theory whatever, particularly if it is ſtill ſubject to doubts and objections. But this the author ſeems to have entirely neglected, by only admitting into his book, ſuch contributions which appear conſiſtent with Brunonian principles; and how little this partiality is able to promote truth and real knowledge, needs no farther demonſtration. It contains *ſome remarks on Brown's theory, a ſketch of the ſyſtem, and relations of ſeveral diſeaſes and caſes*; but on the whole, not any new idea or arguments in ſupport of it; and though it cannot be denied, that, now and then, an intereſting obſervation is met with, this cannot, however, induce us to wiſh, that the author ſhould continue a blow, for which he ſeems not altogether calculated.

---

### *Mr. Scarpa, on the Structure of the Bones.*

THIS celebrated anatomiſt, whoſe great merits and diſcoveries in the fineſt parts of anatomical reſearches are generally acknowledged, has again given a proof of his great abilities and inquiſitive genius, by a publication that appeared laſt year at Leipſic, entitled *De Penitiori Oſſium Structura Commentarius*, 55 pages in 4to. ſplendidly printed, with three plates elegantly engraved. The common opinion hitherto prevailing amongſt anatomiſts was, that bones conſiſted of fibres, plates, or laminæ, lying in different ſtrata one upon another; but this only appears ſo in the bones of children, which, though *improperly*, may be ſaid to be formed of fibres and plates; for, on examining them through a microſcope, they are obſerved to be reticular and ramifying amongſt themſelves; it may therefore generally be aſſerted, that all bones are of a reticular and cellulous texture. To know whether the moſt ſolid, compact, and, as it were, ſtony part of a bone were cellulous, Mr. Scarpa proceeded both in an analytic and ſynthetic way. In repeating Haller's experiments on the incubation of eggs, he diſcovered by the help of a microſcope, in the thigh and ſhin bones, the fineſt reticular texture, which firſt appeared wrinkled, but on the 14th day it repreſented itſelf quite reticular, cellulous, and twiſted like cotton. This he obſerved likewiſe in the bones of an embryo of 28 lines in length. For the analytic inquiſition, he put a human bone into diluted muriatic acid. The earth being diſſolved, and the acid waſhed out, it was macerated in water, and became a woolly reticular texture. The meſhes and interſtices of it are larger in flat and broad bones than in the round ones, cloſer where the bones are compact, and looſer where they are leſs ſolid. The fineſt ſtructure has accordingly a great likeneſs to that of the ſkin. The cellular ſtructure of the bones is particularly obſervable in whales, dolphins, ſharks, and other fiſhes. The following experiments ſeem likewiſe to contradict the common opinion of the laminous laminous ſtructure of the bones: He deſtroyed the ſhin bone of a dog, and forty days after the external rind of it was changed into a ſpongy ſubſtance of the thickneſs of 6 lines, which is the ſame caſe in mortification of bones, where nature ſurrounds the dead end of it with a thick bony ſponge, only by expanding and widening the outer cruſt of the bone. A ſimilar circumſtance happens in the *mollities oſſium* in children, where the bones receive the ſame ſpongy appearance as if they were laid in muriatic acid. A topical morbid mollification is ſometimes obſerved in bones, by which they are changed into a fleſhy maſs, very vaſcular and eaſily bleeding, which evidently ſhews an analogy with common *tela celluloſa*, forming a ſimilar fleſh in ulcers. It is very ſtriking how ſoon the bones of birds produce a ſpongy cotton-like texture when deprived of the perioſteum; "viſu mirabile eſt, in avibus quanta celeritate ex oſſibus de induſtria perioſtes nudatis, mollis carun-cula propullulat; ſanguiferis vaſis plurimum referta, quæ porro in cartilaginem primum, mox intenuiſſimum quoddam oſſeum goſſy-pium ſubtiliter cum extus tum intus reticulatum convertitur." The bones poſſeſs a greater quantity of veſſels than is generally believed, but thoſe that penetrate into them by the *pori Haverſii* do not proceed in a ſtraight direction, but form nets in the internal ſubſtance; they are likewiſe provided with nerves, though theſe cannot be anatomically demonſtrated on account of their ſubtility and cloſe ſituation to the veſſels; but it is ſhown, by experience, that the young fleſh ſhooting out of wounds of the bones is poſſeſſed of great ſenſibility. The external plate of the flat bones of the ſkull appears like diploë, when its internal lamina is already quite ſolid. The formation of cavities in bones ſeems to proceed in a mechanic way, by the hardening of the rind.

